# Effects of High Temperature-Triggered Transcriptomics on the Physiological Adaptability of *Cenococcum geophilum*, an Ectomycorrhizal Fungus

**DOI:** 10.3390/microorganisms10102039

**Published:** 2022-10-15

**Authors:** Tianyi Yan, Panpan Zhang, Wenbo Pang, Xiaohui Zhang, Chunlan Lian, Taoxiang Zhang

**Affiliations:** 1 International Joint Laboratory of Forest Symbiosis, College of Forestry, Fujian Agriculture and Forestry University, Fuzhou 350002, China; 2 Asian Research Center for Bioresource and Environmental Sciences, Graduate School of Agricultural and Life Sciences, The University of Tokyo, Tokyo 188-0002, Japan

**Keywords:** ectomycorrhiza, *Cenococcum geophilum*, high temperature tolerance, succinate secretion, antioxidant enzyme activity

## Abstract

High temperature stress caused by global warming presents a challenge to the healthy development of forestry. *Cenococcum geophilum* is a common ectomycorrhizal fungus (ECMF) in the forest system and has become an important fungus resource with application potential in forest vegetation restoration. In this study, three sensitive isolates of *C. geophilum* (ChCg01, JaCg144 and JaCg202) and three tolerant isolates of *C. geophilum* (ACg07, ChCg28 and ChCg100) were used to analyze the physiological and molecular responses to high temperature. The results showed that high temperature had a significant negative effect on the growth of sensitive isolates while promoting the growth of tolerant isolates. The antioxidative enzymes activity of *C. geophilum* isolates increased under high temperature stress, and the SOD activity of tolerant isolates (A07Cg and ChCg100) was higher than that of sensitive isolates (ChCg01 and JaCg202) significantly. The tolerant isolates secreted more succinate, while the sensitive isolates secreted more oxalic acid under high temperature stress. Comparative transcriptomic analysis showed that differentially expressed genes (DEGs) of six *C. geophilum* isolates were significantly enriched in “antioxidant” GO entry in the molecular. In addition, the “ABC transporters” pathway and the “glyoxylate and dicarboxylic acid metabolic” were shared in the three tolerant isolates and the three sensitive isolates, respectively. These results were further verified by RT-qPCR analysis. In conclusion, our findings suggest that *C. geophilum* can affect the organic acid secretion and increase antioxidant enzyme activity in response to high temperature by upregulating related genes.

## 1. Introduction

With the massive emission of greenhouse gases, global warming is becoming more and more serious [1]. According to record, the period from 2010 to 2019 is the decade with the highest temperature, and global warming continues. Global warming not only affects the water cycle and balance but also inhibits nutrient uptake and photosynthesis of plants, resulting in the inhibition of plant growth, withering and even death [2]. Many studies have shown that high temperature stress caused by global warming has led to a large number of forest death [3]. At present, high temperature has become one of the major abiotic stresses affecting the growth and productivity of trees worldwide.

Ectomycorrhizal fungus (ECMF) is a typical symbiotic fungus, and there are 20,000–25,000 species of fungi that can form an ectomycorrhizal symbiosis with land plants [4,5]. ECMF plays a key role in promoting the growth of host plants by elevating the uptake of water and mineral nutrients, especially phosphorus and nitrogen [6]. Moreover, ECMF symbiosis is generally recognized to have many benefits to forest ecosystems [7], such as maintaining the diversity of forest species and helping plants against abiotic and biotic stresses. At present, the use of ectomycorrhizal symbiosis with trees to enhance the resistance of forests to abiotic stresses has attracted worldwide attention [8,9,10].

Global climate change not only affects plant growth and development but also significantly influences the relative abundance, community composition and function of ECMF [11]. The high temperature can explain the interspecific variations in ECMF and the frequencies of *Suillus* spp. and *Tomentella* sp. increasing in summer [12]. Jl et al. found that the diversity of ECMF in *Korean pine* roots was significantly higher in summer than that in other months [13]. Soil warming exacerbated the changes in the ECMF community, and the accelerated nutrient cycle in the warming plot may be a main reason affecting the diversity of the ECMF community [14]. The study showed that the increase of soil carbon input caused by temperature warming changed the composition of the soil fungal community, the relative abundance of *Cenococcum geophilum* Fr. (*C. geophilum*) increased at 0–10 cm soil depth, and the relative abundance of *Sebacina* and *Boletus* increased at 10–20 cm soil depth [15]. Temperature also changed the colonization and growth of ECMF mycelia. Kilpelainen et al. measured the percentage of ECM root tips in *Populus angustifolia* seedlings at 14, 20 and 26 °C and noticed that higher temperatures inhibited the colonization of ECM fungus in the root system [16]. In addition, the mycelial growth and morphology were changed due to the up-regulated proteins of mycelium energy metabolism processes, amino acids biosynthesis and metabolism, signaling pathway, transport and translation in central metabolic pathways under temperature stress [17].

Ectomycorrhizal fungus can resist abiotic stress by regulating the absorption of nutrients, antioxidative detoxification systems and expression of key genes [18]. Under abiotic stress, the epitaxial mycelium of ECMF can secrete enzymes and organic acids to increase the absorption of Na, Ca, P and K, and promote the growth of mycelium [19,20]. Moreover, the ectomycorrhiza is able to control osmotic pressure and precipitate heavy metal ions in cells by absorbing nutrient ions so as to alleviate salt stress and heavy metal stress [21]. In addition, antioxidative detoxification systems can help the fungus to remove the accumulation of reactive oxygen species (ROS), which is an important mechanism to alleviate abiotic stress [22,23]. The Cd and Cu treatments significantly increased the activities of catalase (CAT), peroxidase (POD), superoxide dismutase (SOD) and ascorbate peroxidase (APX) of *L. sordid* isolate [24]. ECMF can resist abiotic stress by inducing the expression of key genes and proteins. The increased expression of γ-GCS in ectomycorrhizal fungus *Hebeloma cylindrosporum* can promote glutathione production to cope with Cd stress [25].

*Cenococcum geophilum* Fr. (1829) belongs to Ascomycota, Dothideomycetes, Mytilinidiales, and Gloniaceae [26]. *C. geophilum* is a common ECMF in the forest system, which can form ectomycorrhizal roots with a large variety of trees such as *Betulaceae*, *Fagaceae*, *Pinaceae*, etc. [27]. *C. geophilum* can help host plants expand root systems, absorb mineral nutrients and water, promote photosynthesis, and resist environmental stresses such as drought, salinity and high temperature [28,29,30]. Studies have shown that *C. geophilum* can form abundant sclerotia, which are composed of dormant bodies twined by hyphae and can survive in adverse environmental conditions [31,32]. There is rich genetic diversity in *C. geophilum*, and it directly affects its physiological and ecological functions [33]. For example, *C. geophilum* has strong antioxidant enzyme activity and nutrient uptake ability under abiotic stress, and different genotypes have different resistances [29,30,34]. This species also has a good ability to adapt to temperature changes because of the stable acid phosphatase, xylosidase, glucuronidase, cellobiohydrolase, N-acetyl-glucosaminidase and b-glucosidase activities under different temperatures [35]. However, so far, little information is available about the response and mechanism of high temperature on different genotypes of *C. geophilum*. 

In this study, the effects of different temperature treatments on growth, activities of antioxidant enzymes and secretion of organic acids of *C. geophilum* mycelia were measured. In addition, a comparative transcriptome analysis was carried out on the different genotypes of *C. geophilum*. The aim is to identify physiological and transcriptional responses of *C. geophilum* tolerance to temperature stress and its underlying mechanisms. Based on this study, the results would enhance our understanding of heat tolerance mechanisms in ECMF and provide a theoretical basis for mycorrhizal seedling technology in forestry production under global warming.

## 2. Materials and Methods

### 2.1. Strain and Culture Conditions

Three tolerant isolates (ACg07, ChCg28 and ChCg100) and three sensitive isolates (ChCg01, JaCg144 and JaCg202) were screened by previous experiments. ChCg28 and ChCg100 were isolated by the Joint Laboratory of Forest Symbiosis of Fujian Agriculture and Forestry University, and Chcg01, ACg07, JaCg144 and JaCg202 were provided by the Asian Research Center for Bioresource and Environmental Sciences at the University of Tokyo. The location and host of samples are listed in Supplementary Table S1. The procedures of mycelium culture were described previously by Shi [29] and Li et al. [30]. In order to analyze the effect of temperature on the mycelial growth of *C. geophilum*, the fungal blocks with a diameter of 7 mm were selected from the pre-cultured fungal colonies and then cultured on each petri dish (diameter 9 cm) containing 20 mL MMN agar medium with different temperature treatments (25 °C and 30 °C). Three replicates were conducted for each treatment. After 30 days of culture in the dark, the mycelial area of each isolate was measured by X-Plan 380dIII, Ushikata (Kantum Ushikata Co., LTD., Yokohama, Japan) to assess the temperature tolerance. 

### 2.2. Determination of Superoxide Dismutase (SOD), Peroxidase (POD) and Catalase (CAT) in Mycelium

The mycelia pre-cultured in MMN agar medium covered with cellophane were transferred into 50 mL of liquid MMN culture medium and incubated for 30 days, then followed by temperature treatment (25 °C and 30 °C) for 15 days. The mycelia were filtered and washed three times with PBS buffer (pH value is about 7.2–7.4) to remove the culture medium. ELISA kits (China, Enzyme Biotechnology, Shanghai) were used to determine the activities of SOD, POD and CAT in the mycelium of six isolates of *C. geophilum*. The mycelium filtrate was filtered through a 0.45 µm filter membrane for the determination of organic acids. 

### 2.3. Determination of the Types and Content of Organic Acids in Culture Medium

The content of organic acids (oxalic acid, citric acid, succinic acid, propionic acid) in the filtrate mentioned above was analyzed by high performance liquid chromatography (HPLC, Shimadzu LC-2030, Shimadzu Scientific Instruments Inc., Kyoto, Japan). Organic acids were separated on a Titank C-18 column (3 µm, 150 × 3.0 mm, Japan), eluted isocratically at 37 °C by 20 mM phosphoric acid as mobile phase with a flow rate of 0.425 mL/min. The injection volume was 2 µL, and the detection was performed with a UV detector at 220 nm.

### 2.4. RNA-Seq Analysis

The mycelia of *C. geophilum* isolates (ACg07, ChCg28, ChCg100, ChCg01, JaCg144 and JaCg202) were collected from MMN liquid medium by passing through 0.45 µm sterile filters after culturing at 25 °C and 30 °C for 48 h and rinsing the surface by PBS for three times. The total RNA was extracted by the Trizol method, and the extracted RNA was detected by Nanodropnd2000 spectrophotometer to check the concentration and quality of the total RNA [36]. Library construction, quality verification and further paired-end sequencing were performed by Biomarker Technologies (Beijing, China) using an Illumina NovaSeq 6000 platform. The transcriptome results are compared with the designated reference genome (*Cenococcum geophilum* 1.58, https://mycocosm.jgi.doe.gov/Cenge3/Cenge3.home.html, accessed on 1 January 2022) by the HISAT2 software to get Mapped Data. DESeq2 tool [37] was adopted to identify differentially expressed genes (DEGs) between temperature treatment and control groups using the following criteria: *p* value < 0.05 and |log2 (fold change)| ≥ 0.58. Gene Ontology Consortium (GO) analysis was performed using the top GO method based on the Wallenius’ non-central hyper-geometric distribution (*p* < 0.05). All DEGs were tested for Kyoto Encyclopedia of Genes and Genomes (KEGG) pathway enrichment using KOBAS2.0. The FDR correction (*p* < 0.05) was subjected to reduce false positive prediction of enriched GO terms and KEGG pathways.

### 2.5. RT-qPCR Analysis

To confirm the accuracy of the results of the RNA-Seq, we screened two key genes for each *C. geophilum* isolate for validation. The total RNA was reversely transcribed to cDNA using the Eastep^®®^ RT Master Mix Kit (Promega Bio, Shanghai, China) according to the manufacturer’s instructions. The RT-qPCR assay was performed using Taq Pro Universal SYBR qPCR Master Mix (Vazyme, Fuzhou, China) with a CFX Connect Real-Time System (Bio-Rad, USA). This experiment was performed with 3 biological replicates and 3 technical replicates. Step One Real-Time PCR fluorescence quantitative PCR instrument was used to detect the Ct value of each template, and the relative expression changes of the target gene in the control group (25 °C) and the experimental group (30 °C) were calculated by 2^−∆∆Ct^ method [38]. The *C. geophilum* 18S rRNA was used as the reference gene [39]. Specific primer sequences (Table S2) of selected genes were designed with Premier 3 Plus (http://www.premierbiosoft.com/, accessed on 1 July 2022). The primers used for RT-qPCR were synthesized by Fuzhou Yihe Biotechnology Co., Ltd. (Fuzhou, Fujian, China).

Tolerant isolates were validated for genes screened from shared “ABC transport” pathway, namely K441DRAFT_643842 and K441DRAFT_695880 (ACg07); K441DRAFT_663665 and K441DRAFT_672389 (ChCg28); K441DRAFT_587262 and K441DRAFT_702334 (ChCg100). Sensitive isolates were validated for genes screened from shared “glyoxylate and dicarboxylate metabolism” and “fatty acid degradation” pathways, namely K441DRAFT_36643 and K441DRAFT_618447 (ChCg01); K441DRAFT_687784 and K441DRAFT_618447 (JaCg144); K441DRAFT_36643 and K441DRAFT_618447 (JaCg202). In particular, K441DRAFT_618447 is an important gene that all three susceptible isolates have annotated in the “fatty acid degradation” pathway, so ChCg01, JaCg144 and JaCg202 all validate it. Similarly, K441DRAFT_36643 was a key gene annotated in the common pathway “glyoxylate and dicarboxylate metabolism” of ChCg01 and JaCg202 isolates.

### 2.6. Statistical Analysis

Significance differences of mycelial growth areas of each isolate between the high temperature treatment and control were assessed using Student’s *t* test (*p* < 0.05). The significance of differences in organic acid concentrations and antioxidant enzyme activities (SOD, CAT and POD) of six groups was assessed by the Kruskal–Wallis test (*p* < 0.05). Data were shown as mean ± SD of replicates (*n* = 3). All statistical analyses were performed in SPSS 22.0.

## 3. Results

### 3.1. Effects of High Temperature Treatment on Mycelial Growth of C. geophilum

**Figure 1:**
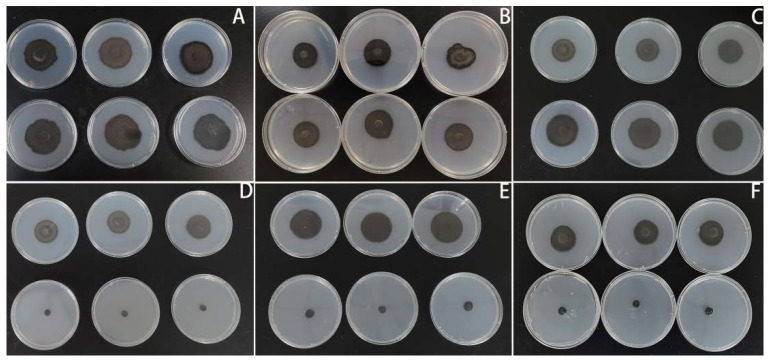
The mycelial growth areas of different isolates of *Cenococcum geophilum* under different temperatures after 30 days culture. In each image, the first row shows the growth area of the isolate cultured at 25 °C and the second row at 30 °C. (**A**) ACg07, (**B**) ChCg28, (**C**) ChCg100, (**D**) ChCg01, (**E**) JaCg144, (**F**) JaCg202.

**Figure 2:**
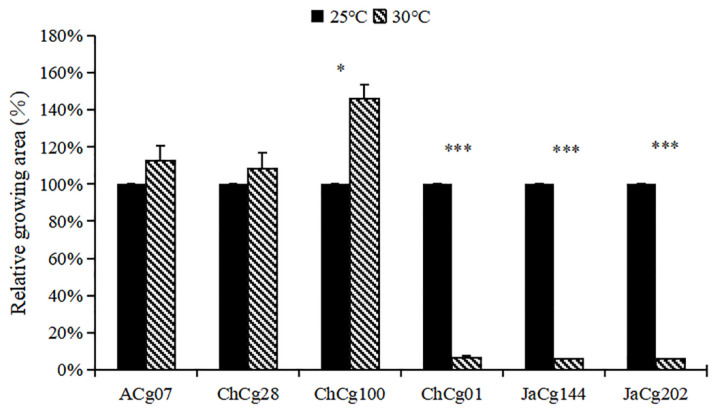
Relative mycelial growth area of the *Cenococcum geophilum* isolates. Relative mycelial area was calculated [(mycelial area at 30 °C)/(mycelial area at 25 °C)] ×100(%). The data showed the mean ± SD for 25 °C and 30 °C replicates (*n* = 3). Significant difference in the relative mycelial growth area of the *Cenococcum geophilum* isolates between 25 °C and 30 °C was tested by the Student’s *t* test (*p* < 0.05). *** *p* < 0.0001, * *p* < 0.05.

As shown in Figure 1, temperature had a significant negative effect on the growth of ChCg01, JaCg144 and JaCg202 (sensitive isolates), and the relative growth areas of these isolates were separately 6.4%, 5.8% and 5.7% under 30 °C treatment. For ACg07, ChCg28 and ChCg100 (tolerant isolates), high temperature promoted their growth with relative growth of 113%, 108% and 146%, respectively (Figure 2).

### 3.2. Effect of High Temperature on the Content of Organic Acids

**Figure 3:**
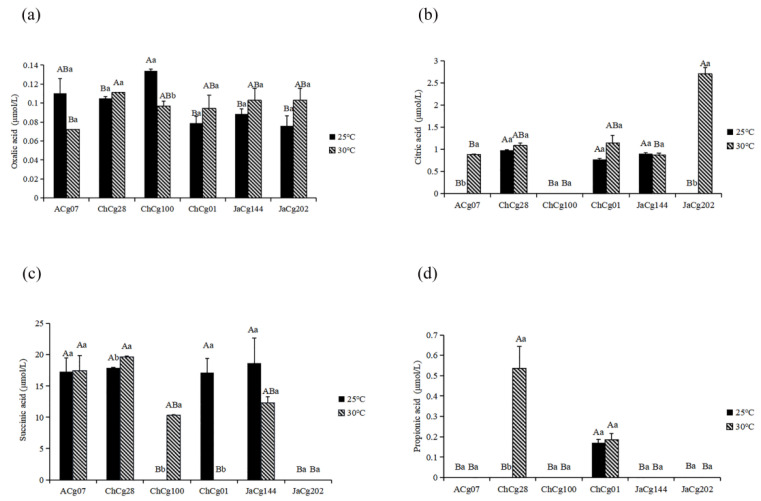
Organic acid concentrations of the six *Cenococcum geophilum* isolates under control and high temperature treatments. (**a**) oxalic acid, (**b**) citric acid, (**c**) succinic acid, (**d**) propionic acid. The data showed the mean ± SD for 25 °C and 30 °C replicates (*n* = 3). Uppercase indicates the difference between different isolates at the same temperature (Kruskal–Wallis test, *p* < 0.05), lowercase indicates the difference between different temperatures of the same isolate (Student’s *t* test, *p* < 0.05).

HPLC UV analysis showed that the organic acids secreted by six *C. geophilum* isolates were mainly composed of succinic acid, oxalic acid, citric acid and propionic acid (Figure 3). Among them, oxalic acid was secreted by all isolates and increased (*p* < 0.05) in three susceptible isolates under high temperature treatment. The secretion content of succinic acid was the highest among the four organic acids. The increased temperature promoted the secretion of succinate by the tolerant isolates, among which the ChCg28 and ChCg100 isolates achieved significant enhance effects (*p* < 0.05). However, high temperature significantly hindered the secretion of succinate by the sensitive isolate ChCg01. Under high treatment, most isolates (ACg07, ChCg28, ChCg01, JaCg202) were promoted to secrete citric acid.

### 3.3. Effect of High Temperature on the Activity of Antioxidative Enzymes

**Figure 4:**
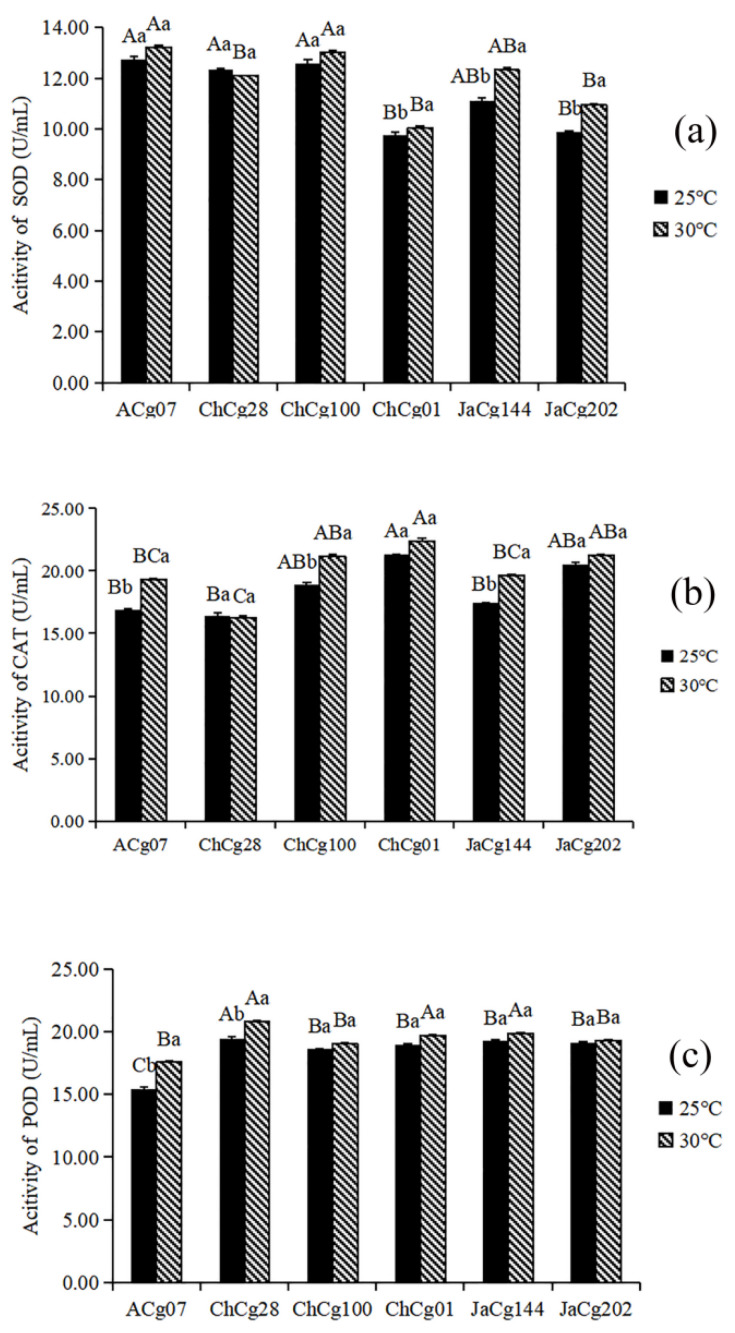
The antioxidant enzyme activities of six *Cenococcum geophilum* isolates under different temperatures. (**a**) SOD, (**b**) CAT and (**c**) POD. The data showed the mean ± SD for 25 °C and 30 °C replicates (*n* = 3). Uppercase indicates the difference between different isolates at the same temperature (Kruskal–Wallis test, *p* < 0.05), lowercase indicates the difference between different temperatures of the same isolate (Student’s *t* test, *p* < 0.05).

Effects of high temperature stress on the activity of antioxidant enzymes of *C. geophilum* are shown in Figure 4a–c. Compared with the control, high temperature stress promoted the activity of POD, SOD and CAT in most isolates (ACg07, ChCg100, ChCg01, JaCg144, JaCg202), and the effect on SOD activity of susceptible isolates reached significant. Most notably, there was no significant difference in POD and CAT activities between sensitive isolates and tolerant isolates (*p* < 0.05), but the SOD activity of tolerant isolates (ACg07 and ChCg100) was significantly (*p* < 0.05) higher than that of sensitive isolates (ChCg01 and JaCg202).

### 3.4. Transcriptome Response of C. geophilum Mycelia in Different Temperatures

#### 3.4.1. Overview of Transcriptome Assembly and Functional Annotation

We sequenced 24 *C. geophilum* RNA-Seq libraries on the Illumina platform and used paired-end sequencing with 150bp at each end. The raw data were submitted to the National Center for Biotechnology Information (NCBI) website, with accession number PRJNA868130. A total of 172.99 Gb clean data was obtained, and each sample clean data reached 6.27Gb. The percentage of Q30 (quality value larger than 99.9%) base was over 92.32%, and 42.43–54.95% of reads were mapped to *C. geophilum* genome in each sample (Table S3). To evaluate the reliability of different biological replicates, Pearson’s correlation coefficient (*R*) examined the correlation of gene expression levels between samples (Figure S2). The *R*^2^ between biological duplicate samples of each isolate is shown in Supplement Table S4, indicating high reliability of library quality. Through species annotations, the results showed that 98.2% of the genes were derived from *C. geophilum* (Figure S1).

#### 3.4.2. Differential Gene Screening

The differentially expressed genes (DEGs) were determined by comparing the FPKM values between 30 °C treatment and control samples in each isolate of *C. geophilum* (Figure S3). Compared to 25 °C, 2740 DEGs were expressed differentially in six isolates under 30 °C treatment, including 1823 down-regulated and 917 up-regulated genes. The number of DEGs of ACg07 (25 °C vs. 30 °C), ChCg28 (25 °C vs. 30 °C), ChCg100 (25 °C vs. 30 °C), ChCg01 (25 °C vs. 30 °C), JaCg144 (25 °C vs. 30 °C) and JaCg202 (25 °C vs. 30 °C) were 223, 535, 787, 752, 168 and 275, respectively, and the up- and down-regulation of DEGs are shown in Supplementary Figure S4.

**Figure 5:**
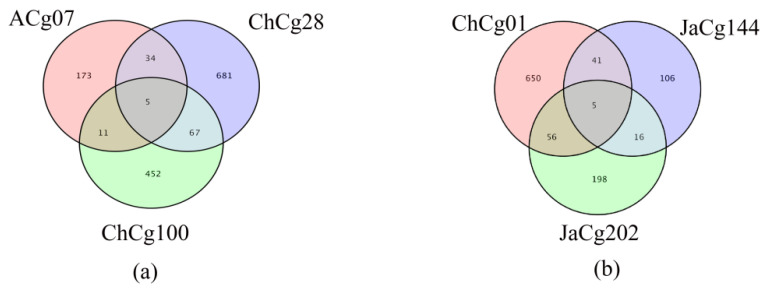
Venn diagram of total DEGs in six isolates of *Cenococcum geophilum* after 30 °C treatment as compared to the same isolates cultured in normal conditions (25 °C). DESeq2_EBSeq, *p* value = 0.05 and |log2 (fold change)| ≥ 0.58 were used as the screening criteria of DEGs. (**a**) three tolerant isolates DEGs, (**b**) three susceptible isolates DEGs.

Venn diagrams were drawn to illustrate the commonality and specificity of these DEGs from different *C. geophilum* isolates. The number of unique genes of ACg07 (25 °C vs. 30 °C), ChCg28 (25 °C vs. 30 °C), ChCg100 (25 °C vs. 30 °C), ChCg01 (25 °C vs. 30 °C), JaCg144 (25 °C vs. 30 °C) and JaCg202 (25 °C vs. 30 °C) were 173, 681, 452, 650, 106 and 198, respectively. The number of common responsive DEGs genes to temperature stress in the sensitive group and tolerant group were both five (Figure 5).

#### 3.4.3. Gene Ontology (GO) Analysis of DEGs

**Figure 6:**
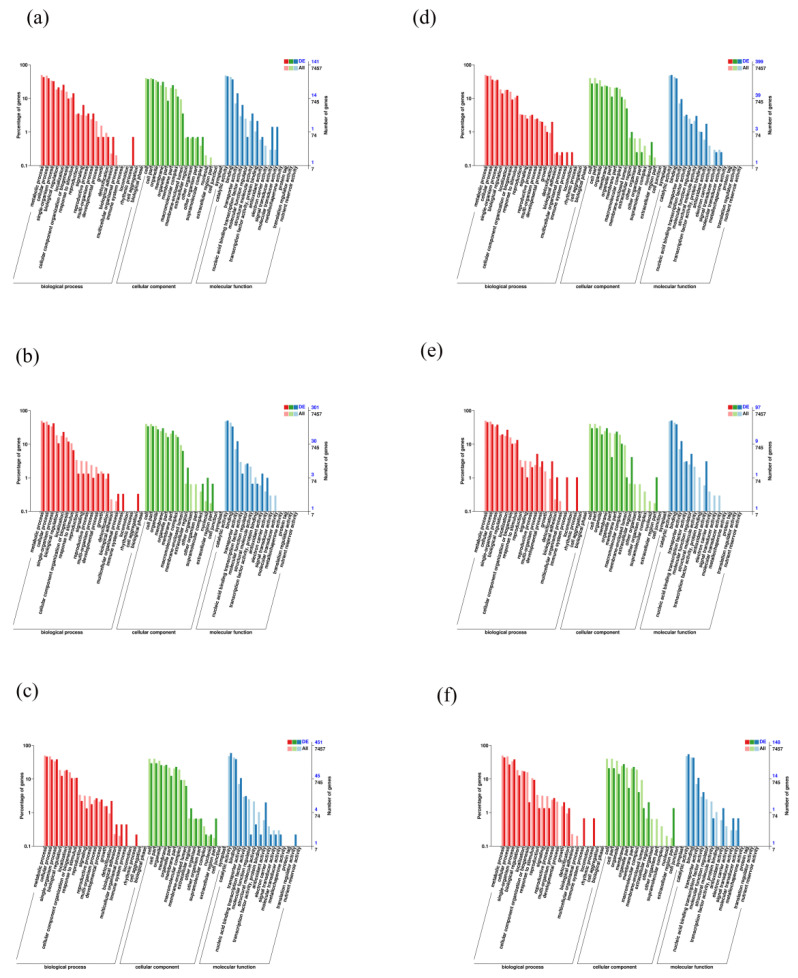
Annotation of GO secondary nodes of differentially expressed genes and all genes in six *Cenococcum geophilum* isolates under temperature stress. (**a**) ACg07 (25 °C vs. 30 °C), (**b**) ChCg28 (25 °C vs. 30 °C), (**c**) ChCg100 (25 °C vs. 30 °C), (**d**) ChCg01 (25 °C vs. 30 °C), (**e**) JaCg144 (25 °C vs. 30 °C), (**f**) JaCg202 (25 °C vs. 30 °C).

GO enrichment analysis showed that the DEGs of six isolates were divided into three categories: biological process, cellular component and molecular function. The top GO items “metabolic processes”, “cellular processes” and “sign-organism process”, which belonged to biological process, “cell”, “cellular parts” and “organelles”, which belonged to the cellular component category, as well as “membranes catalytic activity”, “binding” and “transporter activity”, which belonged to the molecular function category were all enriched in six *C. geophilum* isolates (Figure 6). In addition, the temperature-tolerant group shared the processes of “supramolecular complex”, “transcription factor activity” and “protrin binding”, while temperature-sensitive *C. geophilum* isolates shared little process after 30 °C treatment. More importantly, DEGs of six *C. geophilum* isolates were also significantly enriched in “antioxidant” entry in the molecular function, indicating that these pathways may be important strategies for *C. geophilum* to cope with heat stress (Figure 6).

#### 3.4.4. Kyoto Encyclopedia of Genes and Genomes (KEGG) Analysis of DEGs

**Figure 7:**
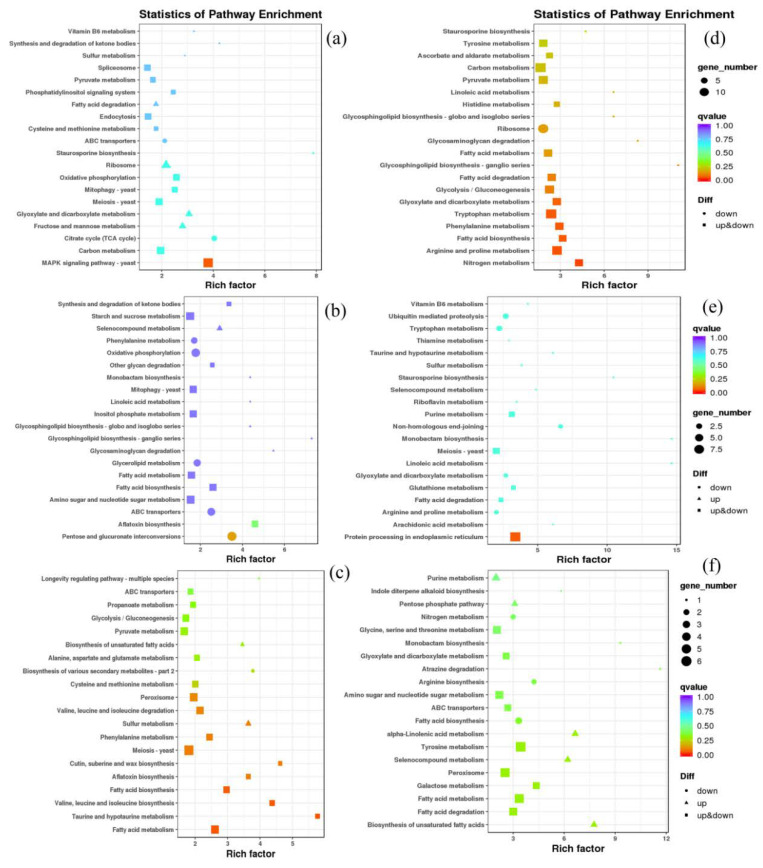
Plot of KEGG pathway enrichment of six *Cenococcum geophilum* isolates up-regulated differentially expressed genes. (**a**) ACg07 (25 °C vs. 30 °C), (**b**) ChCg28 (25 °C vs. 30 °C), (**c**) ChCg100 (25 °C vs. 30 °C), (**d**) ChCg01 (25 °C vs. 30 °C), (**e**) JaCg144 (25 °C vs. 30 °C), (**f**) JaCg202 (25 °C vs. 30 °C).

The KEGG enrichment analysis showed that many different pathways were enriched in the six isolates when treated at 30 °C (Figure 7). The “fatty acid degradation” pathway was found to be significantly enriched in most isolates (ACg07, ChCg01, JaCg144, JaCg202), indicating that fatty acid degradation is an important response mechanism of *C. geophilum* to temperature stress. It is worth mentioning that the three tolerant isolates were significantly shared in “ABC transporters”, while three sensitive isolates were co-enriched in “glyoxylate and dicarboxylate metabolism” pathways. Furthermore, the “Synthesis and degradation of ketone bodies” and “Pyruvate metabolism” pathways were enriched in some tolerant isolates. Some amino acid metabolisms, such as “arginine and proline metabolism”, “tryptophan metabolism” and “tyrosine metabolism” were significantly enriched in some sensitive isolates.

#### 3.4.5. Genes Involved in Key and Related Metabolism Pathways for High Temperature Tolerance and Reduction in *C. geophilum* Isolates 

**Figure 8:**
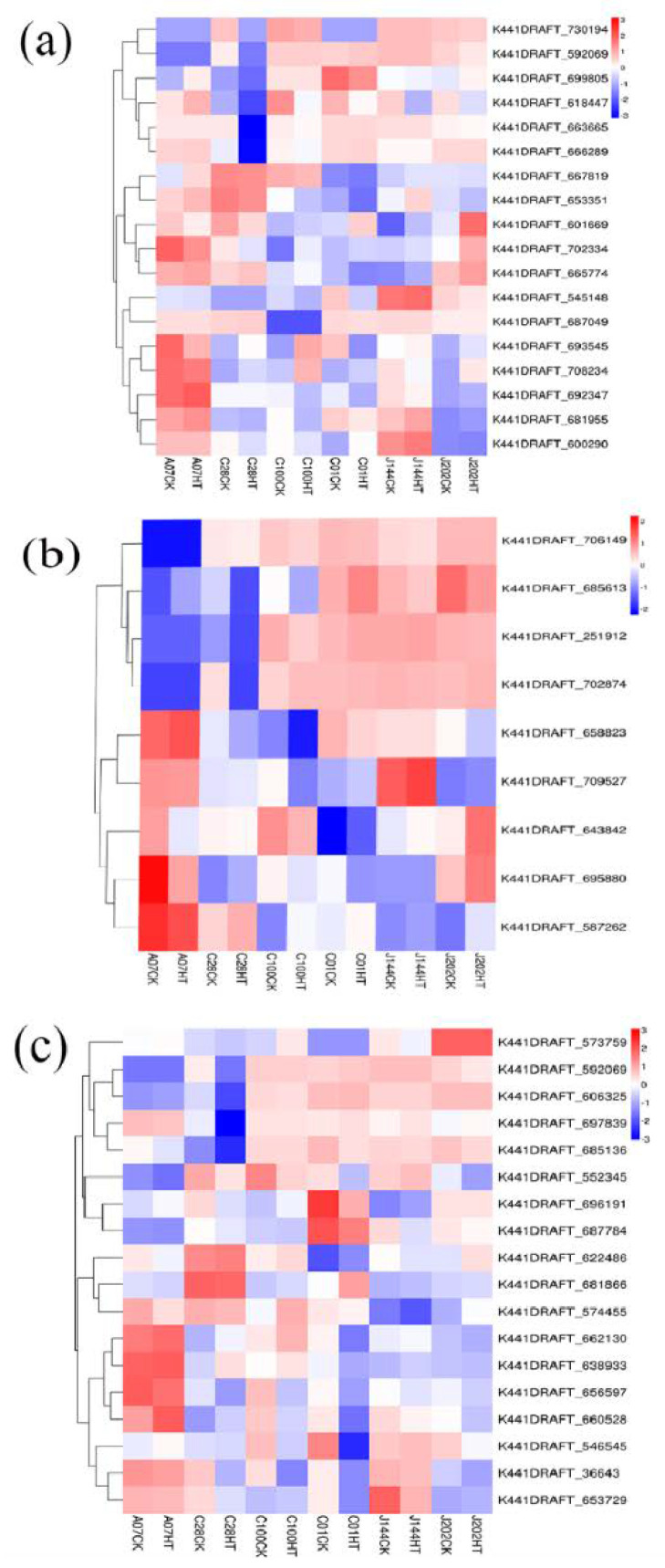
(**a**) Heatmap of genes annotated for the “fatty acid degradation” pathway in six isolates under high temperature treatment, (**b**) heatmap of genes annotated to the “ABC transporter” pathway in three tolerant isolates under high temperature treatment, (**c**) heatmap of genes annotated to the “glyoxylate and dicarboxylate metabolism” pathway in three sensitive isolates under high temperature treatment.

A total of 19 DEGs were significantly enriched in the “fatty acid degradation” pathway (Figure 8a), among which the K441DRAFT_618447 and the K441DRAFT_663665 were shared by the 3 susceptible-isolates and 3 tolerant-isolates, respectively, and the expression levels of both genes decreased under temperature stress (Table S5). For the “ABC transporters” pathway of the temperature-tolerant group, nine of the DEGs were identified (Figure 8b). Among them, we found that K441DRAFT_643842 and K441DRAFT_672389 were both down-regulated in tolerant isolates, while some DEGs (K441DRAFT_695880, K441DRAFT_587262, K441DRAFT_693545, K441DRAFT_702334) were up-regulated in tolerant isolates after temperature stress (Table S5). Additionally, 20 of the DEGs were found in “glyoxylate and dicarboxylate metabolism”, which is the common pathway of susceptible isolates (Figure 8c). Five DEGs (K441DRAFT_36643, gene-K441DRAFT_552345, K441DRAFT_656597, K441DRAFT_660528, K441DRAFT_687784) were down-regulated, and only gene (K441DRAFT_ 681866) was up-regulated after temperature treatment (Table S5). The ATP citrate synthase (K441DRAFT_662130 and K441DRAFT_638933) and phosphoenolpyruvate carboxylic acid kinase (K441DRAFT_574455) involved in oxalic acid decomposition were highly expressed in resistant isolates. The succinate dehydrogenase (K441DRAFT_606325) involved in the breakdown of succinic acid was highly expressed in the sensitive isolates (Figure 8c).

#### 3.4.6. RT-qPCR Verification of DEGs 

**Figure 9:**
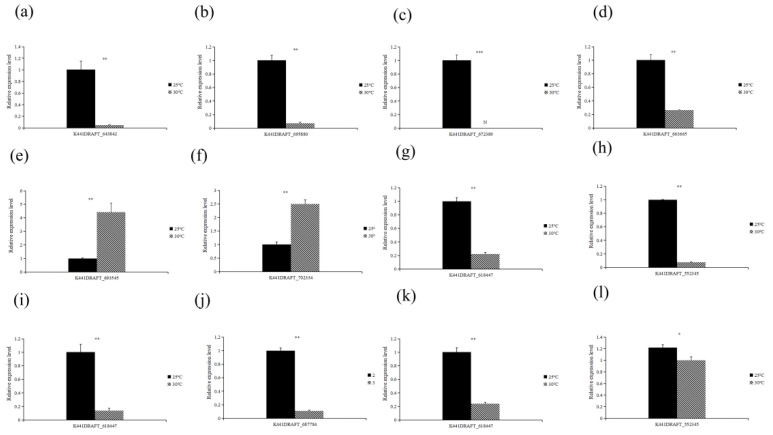
RT-qPCR analysis of the expression level of nine candidate genes in different samples. (**a**,**b**) two key genes verified by ACg07, (**c**,**d**) two key genes verified by ChCg28, (**e**,**f**) two key genes verified by ChCg100, (**g**,**h**) two key genes verified by ChCg01, (**i**,**j**) two key genes verified by JaCg144, (**k**,**l**) two key genes verified by JaCg202. The *Cenococcum geophilum* 18S gene was used as an internal control and gene expression profiles were evaluated using the 2^−ΔΔCt^ method. Three biological replicates and three technical replicates for each sample were performed, and the bars represented the SD of the average mean. Black bars represent relative gene expression under control (25 °C), and the black and white bars represent relative gene expression under treatment (30 °C). The statistically significant difference between the control and high temperature treatment groups (Student’s *t*-test): * *p* < 0.05; ** *p* < 0.01; *** *p* < 0.0001. N represents the gene that is not expressed under 30 °C treatment.

The relative expression of these genes showed that K441DRAFT_587262 and K441DRAFT_702334 were significantly up-regulated, and the rest of the genes were significantly down-regulated under high temperature treatment compared with the control. As shown in Figure 9, transcriptome sequencing results were consistent with the expression profiles of these selected genes, indicating that the DEGs and pathways identified by RNA-Seq were reliable for understanding the response of *C. geophilum* to high temperature stress.

## 4. Discussion

With global warming, the impact of local extreme high temperature on tree health and ecosystem stability has attracted more and more attention. ECMF plays a key role in promoting the growth of host plants and maintaining the stability of forest ecosystems. Previous research [40] showed that high temperature is one of the important abiotic drivers affecting the growth and metabolism of ECMF. *C. geophilum* is a globally distributed ECMF, and China is also rich in *C. geophilum* resources. Research showed that *C. geophilum* has outstanding drought resistance and barren resistance and is often a dominant population under various harsh habitat conditions [35]. Therefore, *C. geophilum* has become an important fungus resource with application potential in forest vegetation restoration. However, the response mechanism of *C. geophilum* to high temperature is not clear. In this study, three temperature-sensitive and three temperature-tolerant *C. geophilum* isolates were selected to analyze their physiological and molecular responses to high temperature stress. We found that the “antioxidant” and “transporter activity” items annotated by GO were two important ways for isolates to resist high temperature. Meanwhile, the activity of SOD, CAT and POD increased under high temperature stress, which also supported this conclusion. The “ABC transporters” pathway is co-enriched by tolerant isolates to cope with high temperature stress. Key genes of “glyoxylate and dicarboxylate metabolism” pathway regulate the secretion of organic acid in response to high temperature stress. 

In the organic acid detection, we found that three sensitive isolates secreted more oxalic acid under temperature stress, while the three tolerant strains secreted more succinic acid. Mycorrhizal fungi can be stimulated to secrete organic acids, which is capable of improving the availability of mineral elements and modifying membrane permeability to enhance their resistance to stress environments [41,42,43]. Oxalic acid is an important organic acid in ECMF [44,45]. However, for some microorganism, oxalic acid, as a virulent substance, can reduce the environmental pH and destroy the plant defense substance [46]. The oxalic acid has negative effects on *Lentinus edodes* mycelium growth, and the KEGG pathway verified that TCA cycle and glyoxylate and dicarboxylate metabolism are important pathways affecting oxalic acid secretion. Succinate is an intermediate product of citric acid cycle (TCA) and glyoxylate and dicarboxylate metabolism, which can provide energy for the cells, alleviate oxidative stress [47], and is an important molecular signal between mitochondria and cells [48]. Succinate and citrate metabolites were considered primarily as important metabolites for heat-stressed granule cells [47]. In our research, we observed that the oxalic acid decomposition genes were highly expressed in resistant isolates, and the decomposition of succinic acid genes was highly expressed in the sensitive isolates. Hence, we speculate that sensitive isolates can increase the secretion of oxalic acid in response to high temperature, while tolerant isolates can reduce the damage of high temperature to cells by increasing succinic acid secretion.

Under abiotic stress, reactive oxygen species (ROS) accumulate in cells and can damage the health of organisms. Therefore, the scavenging of ROS is an important mechanism for organisms to reduce oxidative damage and abiotic stress [49]. Ectomycorrhizal roots were reported to increase the content of active oxygen-scavenging enzymes in mycelium and host plant to alleviate temperature damage [50]. In this study, the SOD, POD and CAT activity of the three sensitive isolates and two tolerant *C. geophilum* isolates were both elevated under 30℃ treatment, and the SOD content of tolerant isolates was significantly higher than that of sensitive isolates. In addition, the proportion of DEGs annotated with “antioxidant” was significantly higher than the proportion of all genes annotated with “antioxidant” in the GO-enriched molecular function category, and the results of the KEGG pathway annotation showed that *C. geophilum* isolates could resist high temperature stress by up-regulating SOD and CAT activity related genes (K441DRAFT_652442, K441DRAFT_697839, K441DRAFT_573759, K441DRAFT_662961, K441DRAFT_650989). Therefore, the increase of antioxidant enzyme activity, especially SOD activity, was an important physiological response mechanism of *C. geophilum* isolates to high temperature stress.

The ABC transporter family can transport nutrients, secondary metabolites and complex conjugates to the central vacuole or release them to the apoplast through ions across various cell membranes, thereby assisting microorganisms in adapting to various stress environments [51,52,53]. ABC transporter is involved in the regulation of potassium transport, thus helping *Chinese rhizobia* cope with an alkaline environment [54]. The transcript levels of ABC in tall fescue roots of mycorrhizal plants were higher than that of uninoculated plants under Ni stress [55]. Kovalchuk et al. detected 35 genes encoding predicted ABC protein in the genome of *C. geophilum*, including 9 ABC B subfamilies, 8 ABC C subfamilies, 2 ABC D subfamilies and 7 ABC G subfamilies [56]. Studies have shown that the undegraded fatty acids were accumulated in cells if long-chain fatty acids cannot be β-oxidation, leading to a series of abnormalities [57]. In our results, three tolerant isolates significantly enriched “ABC transporters” pathway in the KEGG enrichment pathway, and PXA 1/2 (D subfamily) was significantly up-regulated to transport fatty acyl-CoA (K441DRAFT_587262) into and out of peroxisomes under high temperature stress. ABC D family members are located in mitochondria and are involved in the introduction of long-chain fatty acids into peroxisomes to undergo β-oxidation [58]. In addition, PECI (K441DRAFT_667819) involved in fatty acid β-oxidation was significantly up-regulated in tolerant strains. Hence, we speculate that tolerant isolates induced ABC D family to transport long-chain fatty acids for β-oxidation to maintain cell homeostasis and thus cope with high temperature stress. 

This study analyzed the biological mechanisms of different genotypes of *C. geophilum* in response to high temperature stress. Our results clarified the high temperature resistance strategies of *C. geophilum*, and identified a number of candidate genes involved in the regulation of the high temperature stress, which may provide new insights into the stress responses of ECM fungi under high temperature.

## Data Availability

The raw sequencing reads were deposited in the NCBI Bioproject database under the accession number PRJNA868130.

## Supplementary Materials

The following supporting information can be downloaded at: https://www.mdpi.com/article/10.3390/microorganisms10102039/s1, Table S1: Six *C. geophilum* isolates were collected at sites and hosts ; Table S2: Primer sequences used in this study; Table S3: Statistical table for *C. geophilum* sequencing data evaluation; Table S4: The Pearson's correlation of gene expression levels between samples; Table S5: Differentially expressed genes in the significantly enriched pathway of *C. geophilum* in 30 ℃ against control; Figure S1: Gene species distribution map; Figure S2: Replicated correlation heatmap; Figure S3: Volcano plot of DEGs between tolerant and sensitive six *Cenococcum geophilum* isolates under temperature stress; Figure S4: Up-regulation and down-regulation of genes in *Cenococcum geophilum* isolates.
