# Supplementary material for: Effects of High Temperature-Triggered Transcriptomics on the Physiological Adaptability of Cenococcum geophilum, an Ectomycorrhizal Fungus
Source: Microorganisms. 2022 Oct 15;10(10):2039. doi: 10.3390/microorganisms10102039 (PMC9607556; doi:10.3390/microorganisms10102039)
Supplement: Supplementary file 1 [file microorganisms-10-02039-s001.zip › microorganisms-1923513-supplementary.pdf]

Table S1. Six *Cenococcum geophilum* isolates were collected at sites and hosts.

Isloate	LocatiPon	Host
ACg07	Florida, USA	Pinus elliotii
ChCg028	Hainan Province, China	Eucalyptus
ChCg100	Shenyang Province, China	Pinus tabulaeformis
ChCg001	Yunnan Province, China	Pinus yunnanensis
JaCg144	New Island, Japan	Pinus thunbergii
JaCg202	Mount Fuji, Japan	Salix reinii

Table S2. Primer sequences used in this study.

Gene ID	F-primers(5'->3')	R-primers(5'->3')
18S rRNA(Reference gene)	CGGGTAACGGGGAATTAGGG	CACCAGACTTGCCCTCCAAT
K441DRAFT_643842	GCAGGCCATTCTCTGTACAATTC	AAGTAGTCGATCAGTACGTGAGC
K441DRAFT_695880	AGTATTGGTGGTTTGGTCCTCTC	GTACTAGCGGAAGACCATCTCTG
K441DRAFT_672389	CCATCAGTTCTGGCTTTTGTGAG	CGCCAATCTCTGTCATATCTCCA
K441DRAFT_663665	GGTTGGAGATAGAGTTGGCGTTA	GTATTGTTGGAATGTGCCTGGAG
K441DRAFT_693545	TATGTCAGCCACCTCTATCAACG	TACCAAACGTTCCAAGAGTAGGG
K441DRAFT_702334	CTGATGATGTTGTCGTAACCTGC	GCAATGTCTTCAACGAGAGATGG
K441DRAFT_687784	GAACGAAACCTCATCACAAGCAC	GTAGATGAGGATTGGGATAGGCG
K441DRAFT_552345	GATGGGGAGATCTCATTCTGTGG	ATGGGAGATTTCATACCGAGCTG
K441DRAFT_618447	ATCCTACAGTTTTCTCTGAGCCC	GCCATGTCTAAATCAATCTCCGC

Table S3. Statistical table for *Cenococcum geophilum* sequencing data evaluation

	Clean Reads	Clean Bases	Total Reads	Mapped Reads	GC(%)	Q20(%)	Q30(%)
A07CK1	25,139,859.00	7,541,957,700.00	50,279,718.00	25,243,398 (50.21%)	51.51	97.93	94.08
A07CK2	22,737,840.00	6,821,352,000.00	45,475,680.00	22,833,547 (50.21%)	51.45	97.89	93.99
A07HT1	21,964,790.00	6,589,437,000.00	43,929,580.00	22,059,927 (50.22%)	51.54	97.78	93.62
A07HT2	23,849,305.00	7,154,791,500.00	47,698,610.00	24,911,752 (52.23%)	51.51	98.02	94.31
C28CK1	25,098,768.00	7,529,630,400.00	50,197,536.00	25,206,539 (50.21%)	51.49	98.26	94.18
C28CK2	23,153,862.00	6,946,158,600.00	46,307,724.00	23,277,185 (50.27%)	51.49	97.68	94.44
C28HT1	20,743,503.00	6,223,050,900.00	41,487,006.00	21,667,655 (52.23%)	51.52	98.18	94.42
C28HT2	23,684,580.00	7,105,374,000.00	47,369,160.00	23,790,997 (50.22%)	51.57	98.14	94.38
C100CK1	21,023,655.00	6,307,096,500.00	42,047,310.00	21,899,406 (52.08%)	51.58	98.09	94.29
C100CK2	20,890,787.00	6,267,236,100.00	41,781,574.00	21,709,959 (51.96%)	51.6	98.23	94.63
C100HT1	22,256,257.00	6,676,877,100.00	44,512,514.00	24,177,502 (54.32%)	51.54	98.25	94.71
C100HT2	21,243,415.00	6,373,024,500.00	42,486,830.00	23,348,508 (54.95%)	51.58	98.16	94.47
C01CK1	23,648,022.00	7,094,406,600.00	47,296,044.00	22,924,112 (48.47%)	51.67	98	94.75
C01CK2	24,317,503.00	7,295,250,900.00	48,635,006.00	23,436,384 (48.19%)	51.68	98.12	92.94

C01HT1	25,480,913.00	7,644,273,900.00	50,961,826.00	23,907,539 (46.91%)	51.47	98.08	94.6
C01HT2	24,950,256.00	7,485,076,800.00	49,900,512.00	23,795,110 (47.69%)	51.51	98.07	94.45
J144CK1	27,735,567.00	8,320,670,100.00	55,471,134.00	28,461,437 (51.31%)	51.43	98.11	94.42
J144CK2	24,710,710.00	7,413,213,000.00	49,421,420.00	25,159,857 (50.91%)	51.43	98.23	94.68
J144HT1	22,023,053.00	6,606,915,900.00	44,046,106.00	21,411,848 (48.61%)	51.5	97.47	92.61
J144HT2	28,996,295.00	8,698,888,500.00	57,992,590.00	28,872,587 (49.79%)	51.28	98.05	94.31
J202CK1	27,379,922.00	8,213,976,600.00	54,759,844.00	28,944,184 (52.86%)	51.44	98.1	94.35
J202CK2	26,680,293.00	8,004,087,900.00	53,360,586.00	28,323,485 (53.08%)	51.45	98.19	94.51
J202HT1	26,953,787.00	8,086,136,100.00	53,907,574.00	28,512,771 (52.89%)	51.49	98.19	94.56
J202HT2	21,984,601.00	6,595,380,300.00	43,969,202.00	18,655,039 (42.43%)	52.22	97.4	92.32

Clean Reads refers to the total number of pair-end Reads in Clean Data; Clean Bases (bp) refers to the total number of bases in Clean Data; Total Reads refers to the total number of single-ended Reads in Clean Data; Mapped Reads: The number of Reads paired to the reference genome and the percentage of Clean Reads; GC (%) refers to the percentage of G and C bases in Clean Data to the total bases; Q20 (%) refers to the Clean Data quality value Percentage of bases greater than or equal to 20; Q30 (%) refers to the Clean Data quality value Percentage of bases greater than or equal to 30.

Supplementary Table S4 The Pearson's correlation of gene expression levels between samples

	**A07c1**	**A07c2**	**A07h1**	**A07h2**	**C01c1**	**C01c2**	**C01h1**	**C01h2**	**C100c1**	**C100c2**	**C100h1**	**C100h2**	**C28c1**	**C28c2**	**C28h1**	**C28h2**	**J144c1**	**J144c2**	**J144h1**	**J144h2**	**J202c1**	**J202c2**	**J202h1**	**J202h2**
A07c1	1																							
A07c2	.986**	1																						
A07h1	.977**	.977**	1																					
A07h2	.980**	.977**	.994**	1																				
C01c1	.654**	.654**	.673**	.673**	1																			
C01c2	.719**	.717**	.745**	.750**	.950**	1																		
C01h1	.645**	.608**	.657**	.659**	.842**	.860**	1																	
C01h2	.653**	.626**	.673**	.679**	.864**	.917**	.963**	1																
C100c1	.688**	.717**	.713**	.701**	.719**	.779**	.631**	.684**	1															
C100c2	.680**	.712**	.702**	.689**	.714**	.759**	.611**	.656**	.983**	1														
C100h1	.717**	.742**	.729**	.720**	.702**	.760**	.641**	.676**	.959**	.953**	1													
C100h2	.740**	.754**	.750**	.743**	.723**	.776**	.692**	.713**	.936**	.929**	.987**	1												
C28c1	.642**	.660**	.652**	.657**	.776**	.733**	.563**	.580**	.601**	.585**	.608**	.622**	1											
C28c2	.753**	.775**	.768**	.774**	.662**	.735**	.564**	.604**	.708**	.697**	.693**	.692**	.812**	1										

C28h1	.771**	.791**	.787**	.785**	.649**	.716**	.564**	.595**	.735**	.722**	.747**	.749**	.785**	.958**	1									
C28h2	.796**	.800**	.820**	.803**	.631**	.694**	.580**	.590**	.720**	.701**	.731**	.742**	.746**	.890**	.945**	1								
J144c1	.751**	.761**	.753**	.756**	.719**	.765**	.597**	.635**	.802**	.807**	.773**	.758**	.641**	.712**	.706**	.695**	1							
J144c2	.753**	.757**	.754**	.756**	.742**	.798**	.629**	.671**	.828**	.821**	.807**	.794**	.646**	.709**	.715**	.706**	.970**	1						
J144h1	.723**	.725**	.725**	.725**	.664**	.712**	.549**	.584**	.738**	.734**	.721**	.707**	.588**	.642**	.659**	.686**	.959**	.945**	1					
J144h2	.730**	.739**	.733**	.734**	.682**	.736**	.559**	.605**	.779**	.771**	.766**	.745**	.603**	.669**	.685**	.691**	.972**	.964**	.986**	1				
J202c1	.690**	.697**	.702**	.689**	.650**	.708**	.615**	.644**	.804**	.773**	.810**	.808**	.594**	.652**	.697**	.721**	.733**	.761**	.706**	.735**	1			
J202c2	.692**	.708**	.709**	.708**	.664**	.736**	.587**	.643**	.813**	.786**	.800**	.792**	.599**	.681**	.707**	.681**	.776**	.792**	.740**	.774**	.932**	1		
J202h1	.734**	.737**	.752**	.742**	.654**	.725**	.621**	.659**	.820**	.784**	.825**	.825**	.608**	.677**	.725**	.765**	.766**	.787**	.758**	.779**	.954**	.952**	1	
J202h2	.699**	.687**	.702**	.696**	.655**	.711**	.696**	.706**	.773**	.737**	.797**	.816**	.584**	.627**	.678**	.703**	.700**	.730**	.701**	.703**	.895**	.830**	.894**	1

** at.01 level (two-tailed), significant correlation

"c" represents each isolate at 25°C; "h"represents each isolate at 30°C. The Pearson correlation coefficient was made using SPSS 22.0. ** P<0.01.

Table S5 Differentially expressed genes in the significantly enriched pathway of *Cenococcum geophilum* at high temperature against control

GeneID	Annotation	P value	Log2 FC	Regulated	Comparison group
K441DRAFT_663665	ABC transport	0.001186983	-0.820903542	down	C28CK vs C28HT
K441DRAFT_643842	ABC transport	7.81E-19	-0.885496349	down	A07CK vs A07HT
K441DRAFT_695880	ABC transport	4.26038E-11	-0.915315062	down	A07CK vs A07HT
K441DRAFT_587262	ABC transport	5.78E-05	0.607204749	up	C100CK vs C100HT
K441DRAFT_702334	ABC transport	1.18E-08	0.841453895	up	C100CK vs C100HT
K441DRAFT_672389	ABC transport	0.001044313	-0.957545369	down	C28CK vs C28HT

		0.000282605	-1.063971035	down	C01CK vs C01HT
K441DRAFT_618447	fatty acid degradation	8.82623E-11	-1.22978367	down	J144CK vs J1441HT
		6.80E-05	-0.914747731	down	J202CK vs J202HT
K441DRAFT_36643	glyoxylate and dicarboxylate metabolism	0.000459623	-0.715117814	down	C01CK vs C01HT
K441DRAFT_552345	glyoxylate and dicarboxylate metabolism	0.001012355	-0.910459483	down	C01CK vs C01HT
		0.000252193	-0.713656958	down	J202CK vs J202HT
K441DRAFT_656597	glyoxylate and dicarboxylate metabolism	3.96E-05	-0.745311418	down	C01CK vs C01HT
K441DRAFT_660528	glyoxylate and dicarboxylate metabolism	0.000357651	-0.746615528	down	C01CK vs C01HT
K441DRAFT_687784	glyoxylate and dicarboxylate metabolism	1.72E-16	-0.914159343	down	J144CK vs J1441HT
K441DRAFT_681866	glyoxylate and dicarboxylate metabolism	0.004431583	0.609468918	up	C01CK vs C01HT
K441DRAFT_662130	glyoxylate and dicarboxylate metabolism	3.26E-07	-0.792205572	down	A07CK vs A07HT
K441DRAFT_638933	glyoxylate and dicarboxylate metabolism	0.00220095	0.597569712	up	C28CK vs C28HT
		1.03E-13	-0.658163691	down	A07CK vs A07HT
K441DRAFT_574455	glyoxylate and dicarboxylate metabolism	0.001351191	0.853710593	up	C100CK vs C100HT
		1.14E-05	0.876157225	up	J202CK vs J202HT
K441DRAFT_606325	glyoxylate and dicarboxylate metabolism	0.005822254	-0.784910181	down	C28CK vs C28HT

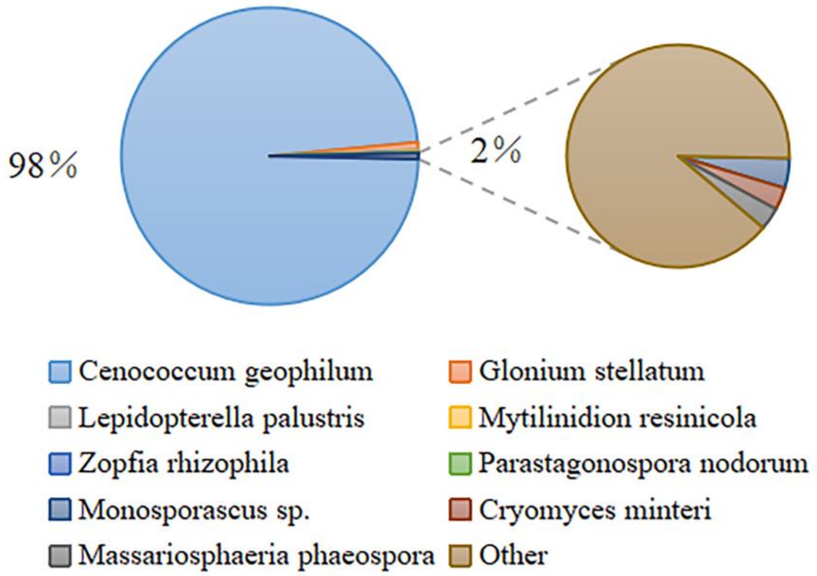

Figure S1. Gene species distribution map

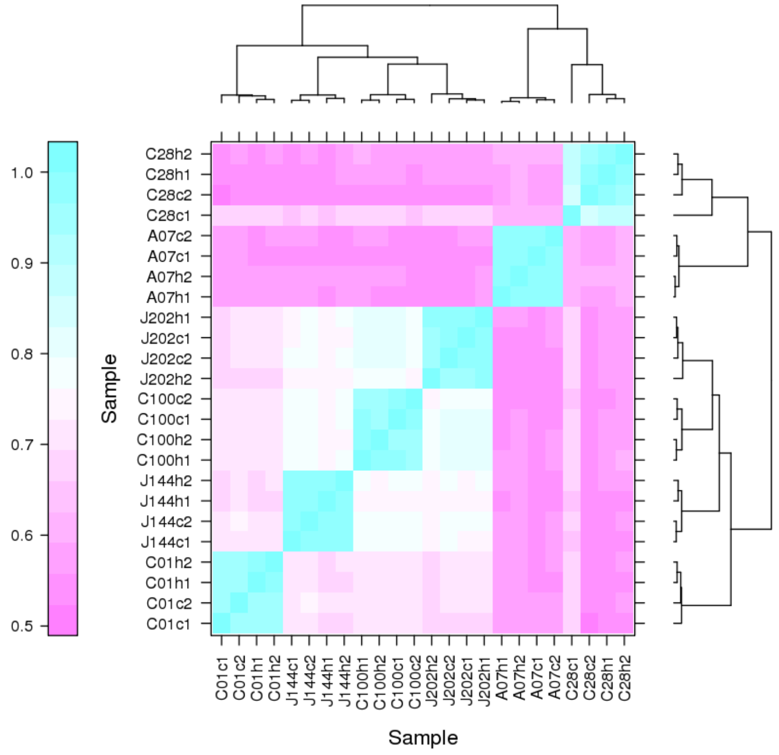

Figure S2. Replicated correlation heatmap

“c” represents each isolate at 25°C; “h”represents each isolate at 30°C; “1” and “2 “represent two biological

repeats of each genotype *Cenococcum geophilum*

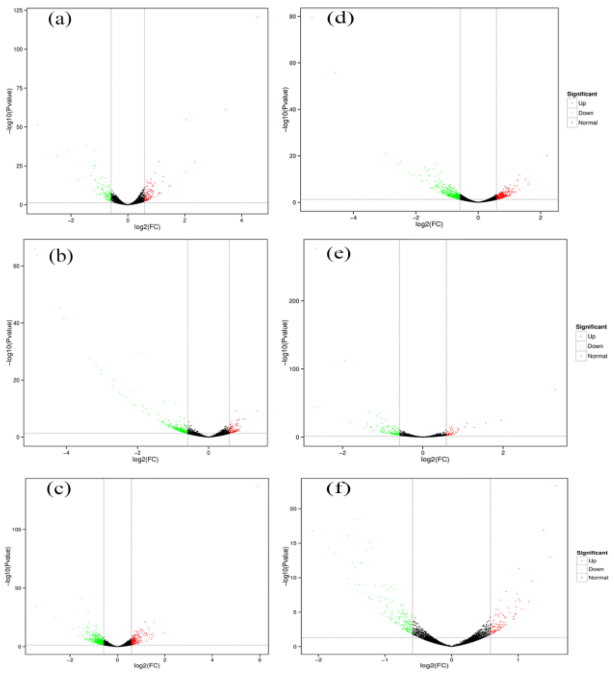

Figure S3. Volcano plot of DEGs between tolerant and sensitive six *Cenococcum geophilum* isolates under temperature stress.

The green dots in the figure represent down-regulated differentially expressed genes, the red dots represent up-regulated differentially expressed genes, and the black dots represent non-differentially expressed genes. (**a**)ACg07 ,(**b**)ChCg28,(**c**)ChCg100, (**d**)ChCg01, (**e**)JaCg144,(**f**)JaCg202. “CK” represents each isolate at 25°C; “HT”represents each isolate at 30°C.

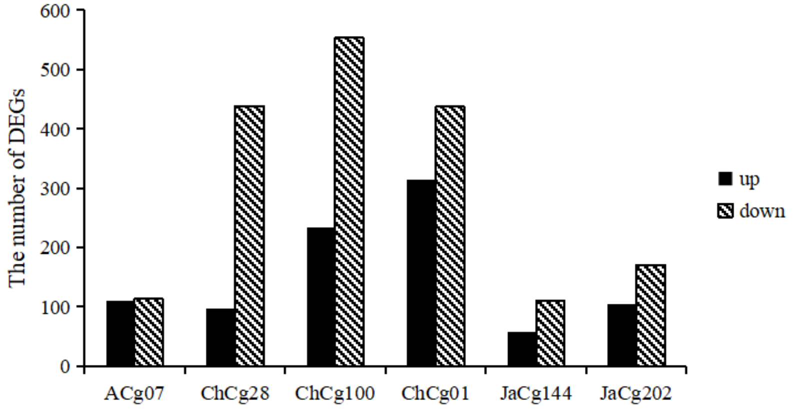

Figure S4. Up-regulation and down-regulation of genes in *Cenococcum geophilum* isolates
